# Uvular Branchial Cleft Cyst with Ectopic Parathyroid

**DOI:** 10.3390/diagnostics15212748

**Published:** 2025-10-30

**Authors:** Anita Sejben, Tamás Lantos, István Sejben

**Affiliations:** 1 Department of Pathology, University of Szeged, 6725 Szeged, Hungary; 2 Department of Medical Physics and Informatics, University of Szeged, 6720 Szeged, Hungary; 3 Department of Pathology, Bács-Kiskun County Teaching Hospital, 6000 Kecskemét, Hungary

**Keywords:** uvula, branchial cleft cyst, ectopic parathyroid

## Abstract

We hereby present a case of a 22-year-old female patient with a lesion on the right side of the uvula. Histopathological assessment identified a cystic lesion with a branching configuration. The cyst lining consisted of non-keratinising stratified squamous epithelium, ciliated columnar epithelium, and focal areas of mucinous columnar epithelium. Within the cystic lumen, a focus of ectopic parathyroid tissue was observed. Based on these findings, a final diagnosis of a uvular branchial cleft cyst containing ectopic parathyroid tissue was established. Pathological lesions of the uvula are rare, and cystic lesions typically correspond to epidermoid cysts. This case, therefore, represents the first documented occurrence of a uvular branchial cleft cyst containing ectopic parathyroid tissue, underscoring the importance of detailed histopathological and immunohistochemical evaluation in the assessment of even small, incidentally detected uvular lesions.

**Figure 1:**
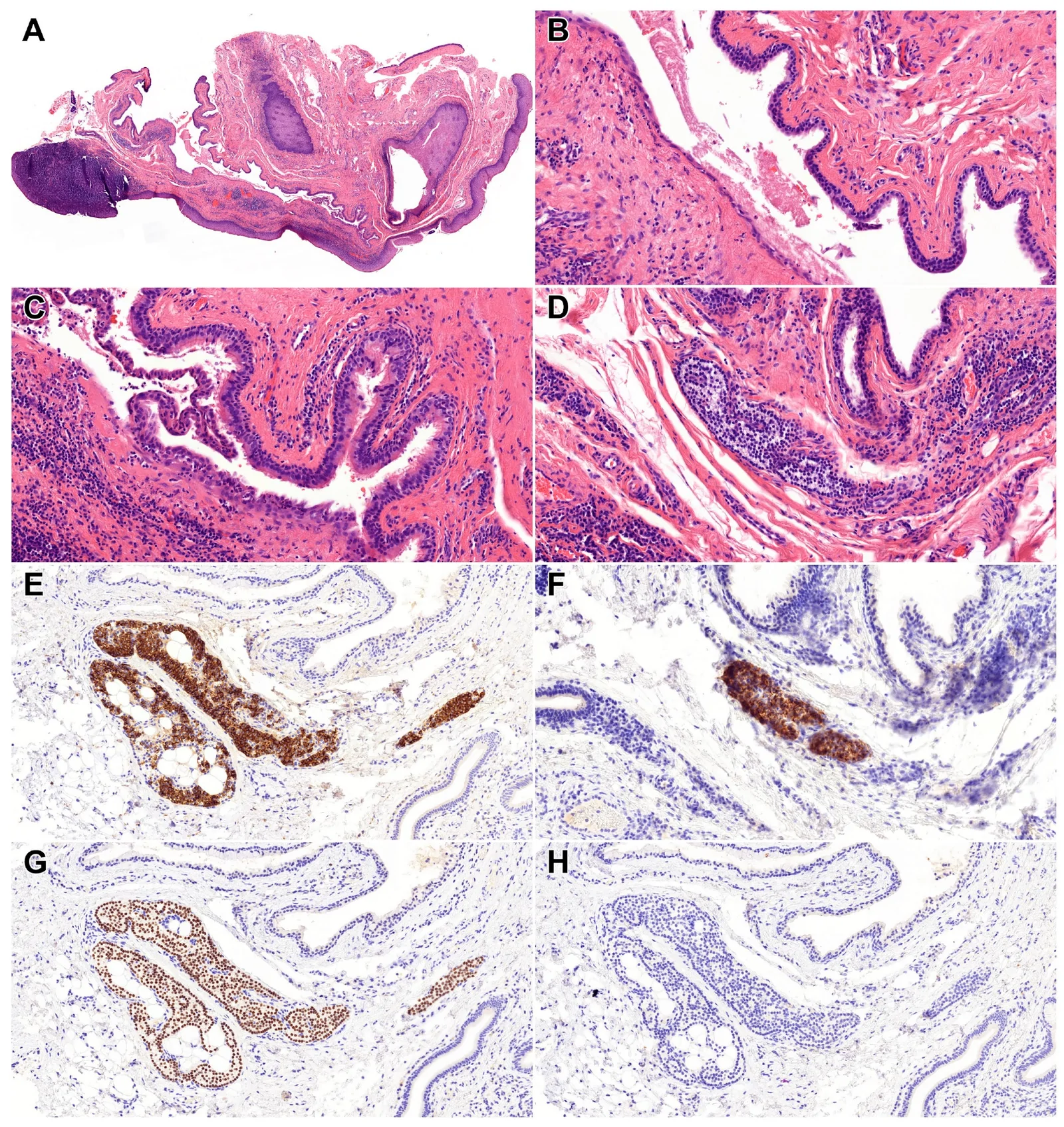
A 22-year-old female patient was admitted to the hospital following the incidental detection of a lesion on the right side of the uvula. The lesion was excised under local anaesthesia, with a provisional diagnosis of a uvular cyst. Comprehensive otolaryngological examination did not reveal any additional pathological findings. Histopathological examination demonstrated an 11 mm cystic lesion exhibiting a branching configuration (**A**—HE, 2×). The cyst was partially lined by non-keratinising stratified squamous epithelium, ciliated columnar epithelium, and focally by mucinous columnar epithelium (**B**,**C**—HE, 20×). The cystic wall contained lymphoid infiltrates with the formation of an organoid lymphoid follicle. Within the cystic wall, ectopic parathyroid tissue was identified, measuring approximately 1 mm in greatest dimension (**D**—HE, 20×). Immunohistochemical staining demonstrated positivity for Chromogranin A (**E**—Chromogranin A, 20×), Parathyroid hormone (PTH) (**F**—Parathyroid hormone, 20×), GATA3 (**G**—GATA3, 20×), and while being negative for TTF-1 (**H**—TTF1, 20×), supporting parathyroid differentiation of the ectopic tissue. Based on the morphological features and immunohistochemical profile, the findings were consistent with a uvular branchial cleft cyst containing ectopic parathyroid tissue. At the 4-month follow-up after diagnosis, the patient remains in good general condition with no evidence of recurrence. Pathological lesions of the uvula are relatively rare in the published literature. Among cystic lesions of the uvula, epidermoid cysts represent the most frequently reported entity, primarily occurring in infants and children [1,2,3]. Branchial cleft cysts of the uvula have not yet been described before. The only notable differential diagnostic challenge in this case was the lymphoepithelial cyst, although both entities share similar therapeutic approaches and clinical outcomes [4]. Uvular cysts may have significant functional implications, including velopharyngeal dysfunction leading to speech impediments, respiratory compromise necessitating emergency intervention, but most commonly remain asymptomatic, being discovered incidentally during routine examination [1,2,3]. While acute uvular swelling typically arises secondary to infectious, allergic, or traumatic events, it may also indicate underlying neoplastic processes, including mucosa-associated lymphoid tissue (MALT) lymphoma or T-cell lymphoma, underscoring the need for comprehensive evaluation [5,6]. Macroscopically irregular or asymmetrical uvular lesions must be distinguished from uvular carcinoma, which is most commonly associated with tobacco exposure and is histologically characterised as squamous cell carcinoma [7]. Interestingly, uvular squamous cell carcinomas have been reported to exhibit a more favourable prognosis compared with other oropharyngeal squamous cell carcinomas [8]. Rarely, uvular manifestations may reflect adjacent or distant neoplastic processes. For example, Wareing et al. described a case of parapharyngeal carcinoma presenting clinically as recurrent uvular oedema [9]. Similarly, Suzuki et al. reported an unusual case of papillary thyroid carcinoma associated with a retropharyngeal cystic goitre extending to the level of the uvula [10]. The presence of ectopic parathyroid tissue is believed to result from aberrant migration during early embryogenesis, reflecting deviations in the normal descent of the parathyroid glands from the third and fourth pharyngeal pouches [11]. Clinically, ectopic parathyroid tissue may be identified due to manifestations of hyperparathyroidism, including hypercalcemia and related metabolic complications. Surgical localisation of ectopic parathyroid glands typically focuses on specific anatomical landmarks. For superior parathyroids, common sites of exploration include the upper thyroid pole and the upper vascular thyroid stalk posterior to the hypopharynx and cervical oesophagus. In contrast, inferior parathyroids are most frequently sought near the carotid artery bifurcation and within the thymic tongue [11,12]. Less commonly, ectopic parathyroid tissue may be encountered in mediastinal or paraaortic locations [12,13,14]. Preoperative identification of ectopic parathyroid lesions can be further enhanced using fine-needle aspiration cytology (FNAC). When performed on a lesion suspected to contain parathyroid tissue, FNAC combined with measurement of PTH levels in the aspirated material allows for more accurate localisation and facilitates surgical planning [11]. Although rare, ectopic parathyroid adenomas and carcinomas have also been documented, underscoring the importance of careful evaluation and consideration of neoplastic potential during both diagnosis and surgical management [15,16]. A review of the current literature revealed no prior documentation of a uvular branchial cyst or uvular ectopic parathyroid tissue, neither was their cooccurrence. To date, there are no previously reported cases of a branchial cleft cyst arising in the uvula, making this an exceptional anatomical presentation. Such heterogeneity is unusual even in typical branchial cleft cysts, let alone in this novel location. Although incidental and asymptomatic, this case broadens the differential diagnosis of uvular lesions. It also emphasises the importance of comprehensive histopathological evaluation, as rare ectopic tissues may be overlooked otherwise.

## Data Availability

The original contributions presented in this study are included in the article. Further inquiries can be directed to the corresponding author.

## Abbreviations

The following abbreviations are used in this manuscript:

FNAC	Fine needle aspiration cytology
GATA3	GATA binding protein 3
HE	Hematoxylin and eosin
MALT	Mucosa-associated lymphoid tissue
PTH	Parathyroid hormone
TTF1	Thyroid transcription factor-1
